# Advancements in cancer immunotherapies targeting CD20: from pioneering monoclonal antibodies to chimeric antigen receptor-modified T cells

**DOI:** 10.3389/fimmu.2024.1363102

**Published:** 2024-04-04

**Authors:** Agnieszka Dabkowska, Krzysztof Domka, Malgorzata Firczuk

**Affiliations:** ^1^ Laboratory of Immunology, Mossakowski Medical Research Institute Polish Academy of Sciences, Warsaw, Poland; ^2^ Department of Immunology, Medical University of Warsaw, Warsaw, Poland

**Keywords:** CD20, B cell, leukemia, lymphoma, immunotherapy, monoclonal antibody, antibody-drug conjugate (ADC), CAR-T

## Abstract

CD20 located predominantly on the B cells plays a crucial role in their development, differentiation, and activation, and serves as a key therapeutic target for the treatment of B-cell malignancies. The breakthrough of monoclonal antibodies directed against CD20, notably exemplified by rituximab, revolutionized the prognosis of B-cell malignancies. Rituximab, approved across various hematological malignancies, marked a paradigm shift in cancer treatment. In the current landscape, immunotherapies targeting CD20 continue to evolve rapidly. Beyond traditional mAbs, advancements include antibody-drug conjugates (ADCs), bispecific antibodies (BsAbs), and chimeric antigen receptor-modified (CAR) T cells. ADCs combine the precision of antibodies with the cytotoxic potential of drugs, presenting a promising avenue for enhanced therapeutic efficacy. BsAbs, particularly CD20xCD3 constructs, redirect cytotoxic T cells to eliminate cancer cells, thereby enhancing both precision and potency in their therapeutic action. CAR-T cells stand as a promising strategy for combatting hematological malignancies, representing one of the truly personalized therapeutic interventions. Many new therapies are currently being evaluated in clinical trials. This review serves as a comprehensive summary of CD20-targeted therapies, highlighting the progress and challenges that persist. Despite significant advancements, adverse events associated with these therapies and the development of resistance remain critical issues. Understanding and mitigating these challenges is paramount for the continued success of CD20-targeted immunotherapies.

## Introduction

**Figure 1:**
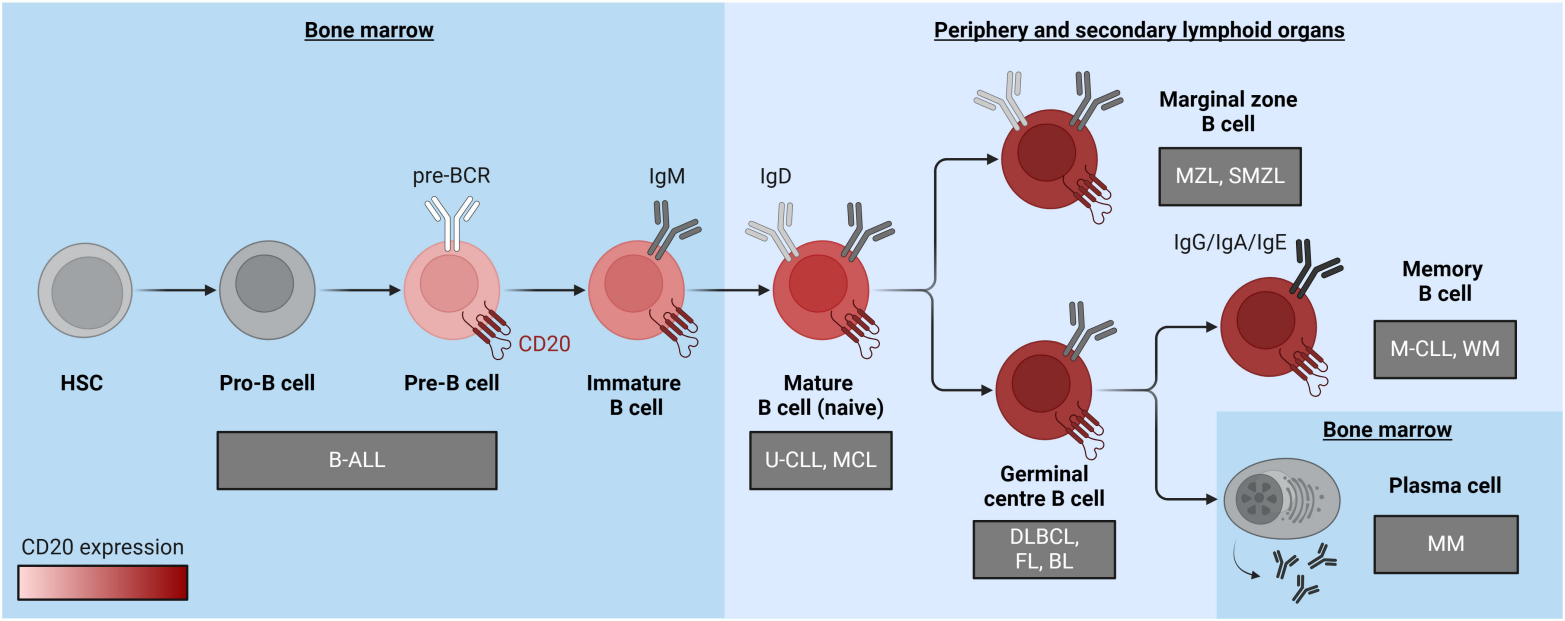
A diagram illustrating B cell differentiation and maturation, emphasizing the pronounced increase in CD20 expression levels depicted through a red color gradient. Associated malignancies are positioned near the cell of origin and represented within grey boxes. B-ALL, B cell acute lymphoblastic leukemia; BL, Burkitt lymphoma; DLBCL, diffuse large B cell lymphoma; FL, follicular lymphoma; HSC, hematopoietic stem cell; MCL, mantle cell lymphoma; M-CLL, mutated chronic lymphocytic leukemia; MM, multiple myeloma; MZL, marginal zone lymphoma; SMZL, splenic marginal zone lymphoma; U-CLL, unmutated chronic lymphocytic leukemia; WM, Waldenstrom macroglobulinaemia. The figure was created using BioRender.com.

CD20 is a surface protein that exhibits ubiquitous expression in B cells with minimal occurrence in other tissues, rendering it an ideal target for immunotherapy against B cell-derived malignancies. CD20 expression initiates during the pre-B cell stage and persists until B cells undergo terminal differentiation into plasma cells (**Figure 1**). Immunotherapy directed at CD20 is extensively employed for treating mature B cell-derived malignancies, such as chronic lymphocytic leukemia (CLL) and various B cell-derived non-Hodgkin lymphomas (B-NHL), including follicular lymphoma (FL), diffuse large B-cell lymphoma (DLBCL), and mantle cell lymphoma (MCL). CD20 is also present in multiple subtypes of B cell precursor acute lymphoblastic leukemia (B-ALL), albeit its expression at diagnosis is heterogeneous and frequently low (1–3). Notably, documented upregulation of CD20 after induction treatment suggests a potential expansion of CD20-directed immunotherapy applications for B-ALL (4, 5). CD20-specific therapies offer precise B cell targeting, minimizing impact on other cell types. These therapies efficiently deplete CD20-expressing B cells without hindering the replenishment of the B-cell compartment from early B cell precursors. Hence, upon cessation of anti-CD20 treatment, the B-cell population can recover (6). Notably, the absence of CD20 on fully mature plasma cells enables patients to maintain protective humoral immunity against previously encountered pathogens during treatment (6).

CD20-targeted immunotherapy encompasses diverse modalities administered at various treatment stages. Rituximab, the pioneering anti-CD20 monoclonal antibody (mAb) introduced in 1997, stands out as a well-studied, low-toxicity immunotherapy with manageable side effects. It is a crucial component of the common therapy regimens, such as BR (bendamustine + rituximab) or FCR (fludarabine + cyclophosphamide + rituximab), which are often used as a first-line treatment in specific groups of CLL and B-NHL patients. In addition, following positive phase 3 trial results, rituximab has been recently integrated into chemotherapy for adult B-ALL patients with at least 20% CD20-positive leukemic cells (7). Beyond rituximab, the engineered anti-CD20 mAb obinutuzumab is registered and employed in combination with chemotherapy as first-line therapy for defined cases of CLL and FL. In addition to mAbs, new immunotherapies targeting CD20 have been developed and successfully introduced into the clinic for patients refractory to first-line therapy or with relapsed disease (r/r). These include bispecific antibodies (BsAbs) targeting the CD20 molecule and simultaneously recruiting cytotoxic T cells, as well as adoptive therapies using autologous T cells modified with chimeric antigen receptors (CAR-T). Three BsAbs targeting CD20 have received FDA approval, while CD20-specific CAR-T cells are presently undergoing clinical trials. Notably, CAR-T cells simultaneously targeting CD19 and CD20 aim to address CD19-negative clones, with ongoing clinical trials in advanced r/r B-cell malignancies (**Table 1**).

**Table 1:** Clinically tested CD20-targeting CAR-T therapies.

Name	Effector cells	Structure	Indications	Clinical trial phase	Clinical trial identifier
CD20 CAR-T	autologous T cells	CD20 scFv with CD8a H/TM, 4-1BB, CD3ζ domains	r/r B-NHL	phase I	NCT04036019
CD19/CD20 CAR-T	autologous T cells	CD20 and CD19 scFv with CD8a H/TM, 41BB, CD3ζ domains	r/r B-cell malignancies	phase I/II	NCT03097770
CD20/CD22 CAR-T	allogeneic T cells	CD20 and CD22 scFv with CD8α H/TM, 41BB, CD3ζ domains	r/r B-NHL	phase I/II	NCT05607420
CD19/CD20/CD22 CAR-T	autologous T cells	CD19, CD20 and CD22 scFv with CD8α H/TM, 41BB CD3ζ domains	r/r B-NHL	phase I	NCT05418088

**Figure 2:**
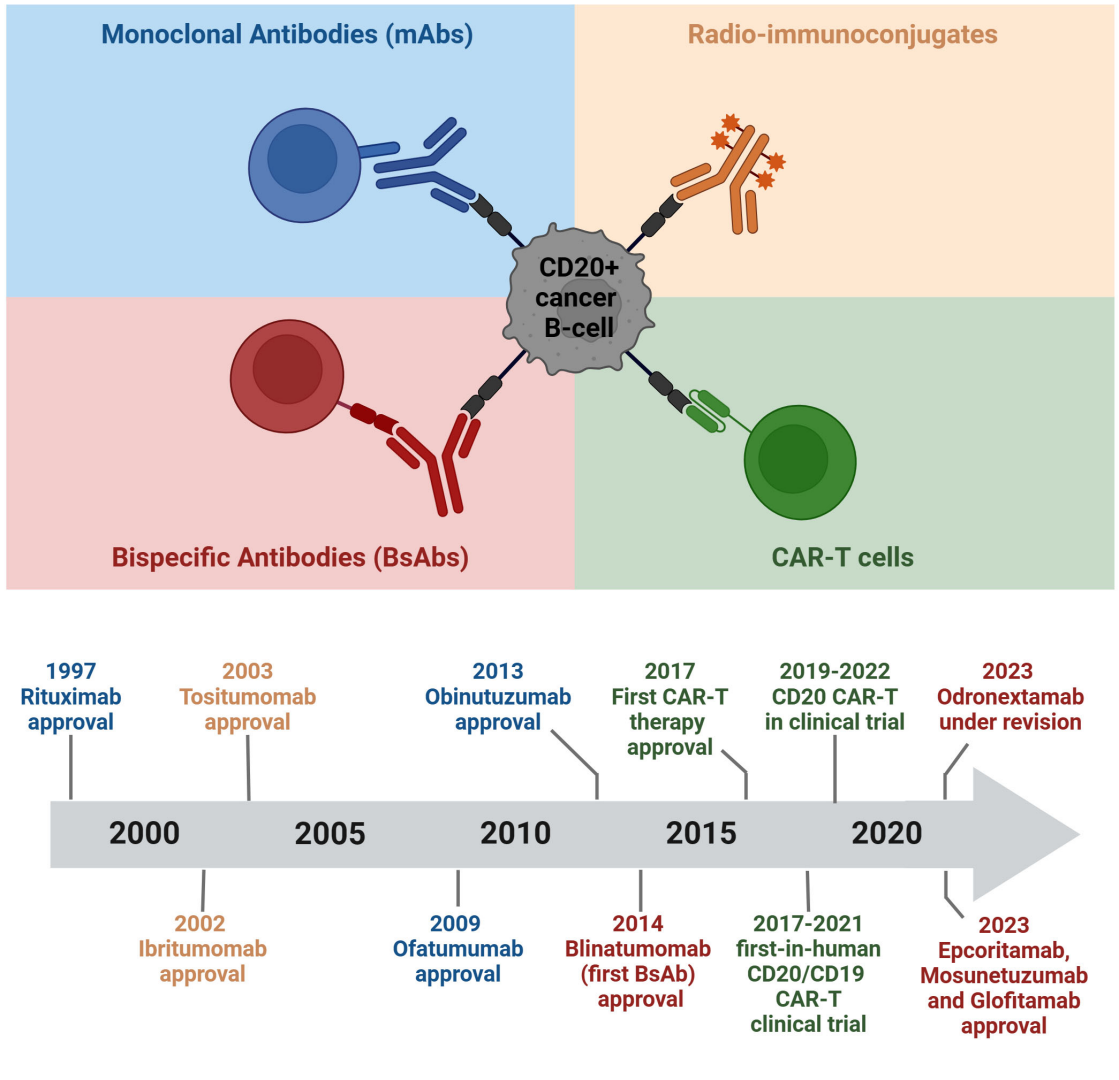
Chronology of clinical approvals and recent breakthroughs in CD20-targeted immunotherapies. The figure was created using BioRender.com.

**Figure 3:**
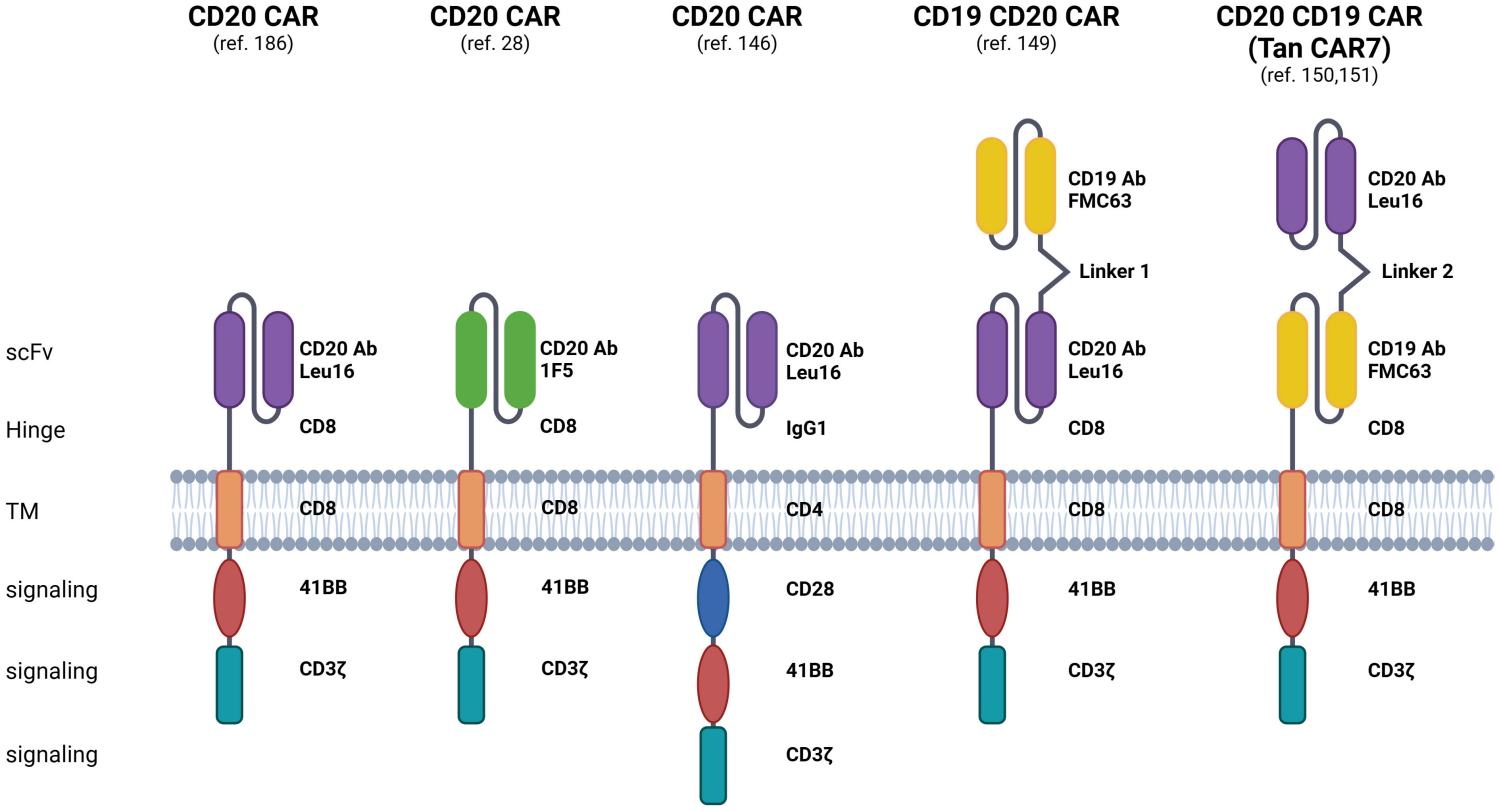
Displayed are CAR constructs, including second- and third-generation designs specifically recognizing the CD20 molecule, alongside dual-specificity CAR constructs recognizing both CD20 and CD19 molecules. Additionally, preclinical and clinical investigations explore the efficacy of bi- or tri-cistronic vectors for the expression of these CARs. Linker 1: (G4S)5, Linker 2: (EAAAK)3. The figure was created using BioRender.com.

**Figure 4:**
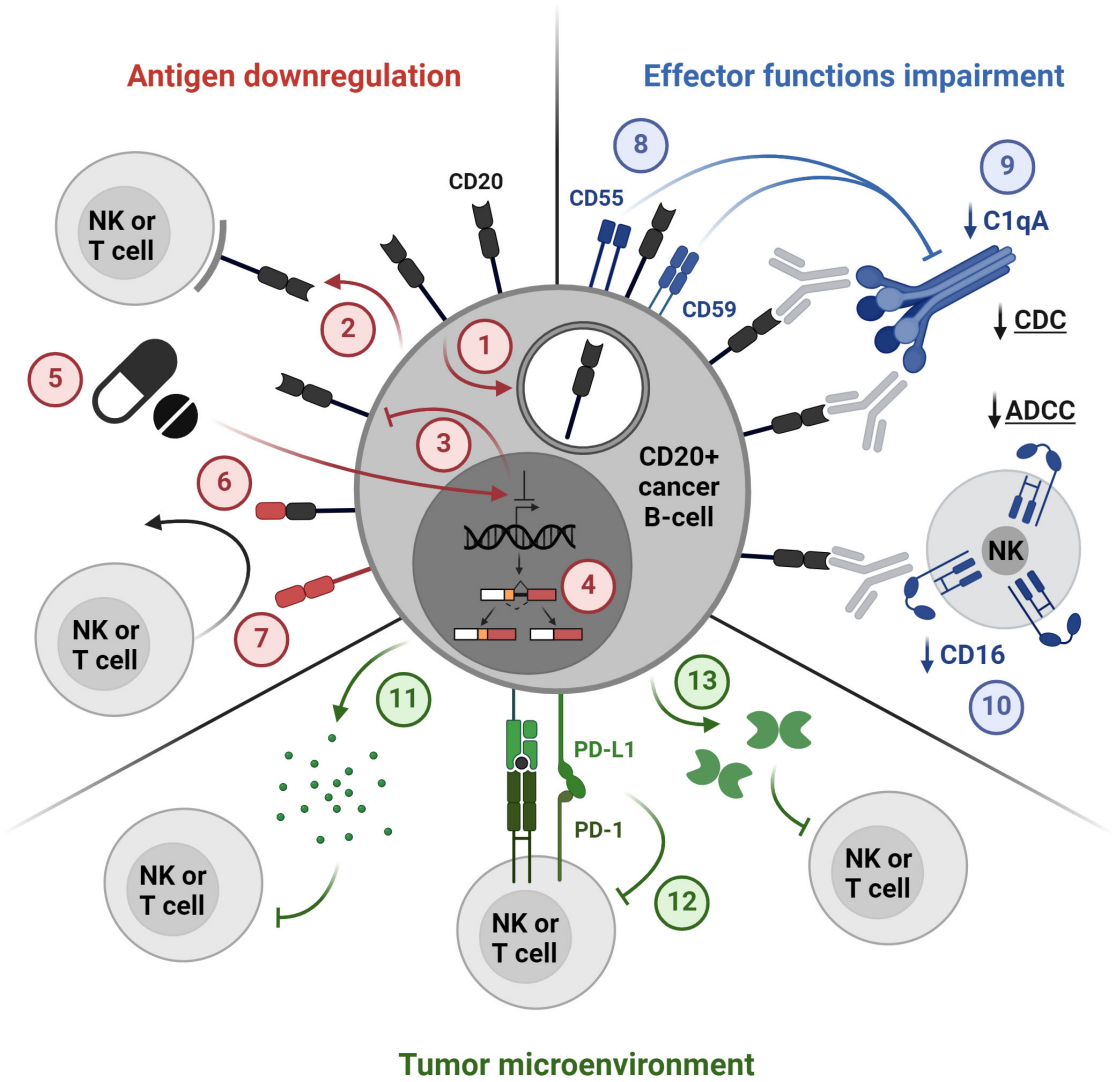
Mechanisms of resistance to CD20-directed immunotherapies. 1. Internalization, 2. Trogocytosis, 3. Loss of antigen expression, 4. Alternative splicing, 5. Drug-induced antigen downregulation, 6. Loss of an epitope, 7. Lineage switch, 8. Overexpression of complement regulatory proteins, 9. Downregulation of complement proteins, 10. CD16 downregulation, 11. Secretion of immunosuppressive cytokines, 12. Immune checkpoints, 13. Secretion of suppressive molecules, e.g. galectin 1. The figure was created using BioRender.com.

This comprehensive review explores various CD20-directed immunotherapies, including mAbs, radio-immunoconjugates, BsAbs, and CD20 CAR-T cells. The discussion encompasses both approved drugs and novel solutions undergoing investigation in preclinical and clinical trials (**Figures 2**, **3**; **Tables 1**–**3**). Mechanisms of resistance to CD20-directed immunotherapies are presented (**Figure 4**), and the potential for various combinations with immunotherapies is discussed.

**Table 2:** Clinically approved and tested anti-CD20 mAbs and radio-immunoconjugates.

Name	Structure	Origin	CD20 epitope characteristics	Fc domain	Approval date*	Indications	Common dosages in clinical setting	Clinical trials
Rituximab	IgG1κ	chimeric	A_(170)_NPS_(173)_ region on large extracellular loop	unmodified	1997	CLL, DLBCL, FL	375 mg/m2 per infusion	PMID: 9310469
Ofatumumab	IgG1	human	FLKMESLNFIRAHT region on large extracellular loop and A74T, I76A, Y77S residues on small extracellular loop	unmodified	2009	CLL	300-2000 mg per infusion	NCT00092274
Obinutuzumab	glycoengineered IgG1κ	humanized	residues 172–176 on large extracellular loop	reduced fucosylation of Fc region	2013	CLL, r/r FL	100-1000 mg per infusion	NCT22431570
Ublituximab	glycoengineered IgG1κ	chimeric	residues 168–171 and 158–159 on large extracellular loop	reduced fucosylation of Fc region	not approved	CLL	≤150 - 900 mg per infusion	NCT02301156
Ocaratuzumab	glycoengineered IgG1	humanized	A_(170)_NPS_(173)_ region on large extracellular loop. Increased affinity to CD20	reduced fucosylation of Fc region; protein-engineered to improve affinity to 158-F FcγRIIIa carriers	not approved	r/r FL	375 mg/m2 per infusion	NCT00354926
90Y-Ibritumomab Tiuxetan	90-yttrium labeled IgG1κ	murine	A_(170)_NPS_(173)_ region on large extracellular loop	unmodified	2002	FL, r/r NHL	14.8 MBq/kg	PMID: 12777454
131I-Tositumomab	131-iodium-linked IgG2aλ	murine	A_(170)_NPS_(173)_ region on large extracellular loop	unmodified	2003	r/r NHL (withdrawn)	75 cGy	PMID:15689582 PMID:11579112

(*) regarding approval in oncological indications.

Information about CD20 epitopes recognized by subsequent Abs is described in (8).

**Table 3:** Clinically approved and tested CD20xCD3 BsAbs.

Name	Structure	Antigen binding domain	Fc domain	Production	Approval date	Indications	Common dosages in clinical setting	Clinical trials
Epcoritamab	full-length IgG1	1 anti-CD20 Fab 1 anti-CD3 Fab	FcγR and C1q binding abolished FcRn binding maintained	controlled Fab arm exchange	2023	r/r DLBCL	0,16-48 mg s.c. in 28-day cycles with step-up dosing	NCT03625037
Mosunetuzumab	full-length IgG1	1 anti-CD20 Fab 1 anti-CD3 Fab	FcγR binding abolished FcRn binding maintained	knobes-into-holes	2023	r/r FL	1-60 mg i.v. in 21-day cycles	NCT02500407
Glofitamab	full-length IgG1	2 anti-CD20 Fab 1 anti-CD3 Fab	FcγR and C1q binding abolished FcRn binding maintained	head to tail fusion via flexible linker	2023	r/r DLBCL	2,5-30 mg i.v. in 21-day cycles with step-up dosing and obinutuzumab pretreatment	NCT03075696
Odronextamab	full-length IgG4	1 anti-CD20 Fab 1 anti-CD3 Fab	FcγRIII binding abolished FcRn binding maintained	heavy chains with different affnities and common light chains	Review	r/r DLBCL, r/r FL	0,1-320 mg i.v. in 21-day cycles with step-up dosing	NCT02290951 NCT03888105
Imvotamab	IgM	10 anti-CD20 Fabs 1 anti-CD3 scFv	unmodified	IgM platform with recombinant J-chain	Not approved	r/r DLBCL, r/r FL	15-300 mg i.v. in 21-day cycles with step-up dosing	NCT04082936
Plamotamab	IgG1	1 anti-CD20 Fab 1 anti-CD3 scFv	FcγR binding abolished FcRn binding maintained	Fab-scFv-Fc format	Not approved	r/r DLBCL, r/r FL	dose-escalation study	NCT02924402

## CD20 antigen: structure, function, and expression regulation

CD20 is a transmembrane protein whose significance as a target for immunotherapy is well recognized, although its biological role remains elusive. Encoded by the *MS4A1* gene, CD20 is part of the MS4A family, which consists of 18 proteins with similar structures. The CD20 protein spans the cell membrane with four transmembrane helices and features two extracellular loops, which are the main epitopes recognized by anti-CD20 mAbs. Notably, both the N-terminal and C-terminal ends of CD20 are situated inside the cell. The detailed structure and dimeric assembly of CD20 on the cell membrane have recently been extensively characterized (9). This structural research sheds light on the molecular architecture of CD20 and anti-CD20 mAbs biding modes, contributing to our understanding of its potential as a target for immunotherapy (9).

While no identified physiological ligand binds to CD20, it is known to form nanoclusters on the B cell membrane with proteins such as IgD or IgM-class B cell receptors (BCR), CD19, CXCR4, and CD40 (10). A recent study utilizing CRISPR/Cas9-mediated CD20 elimination from mature B cells revealed CD20’s role as a gatekeeper in maintaining the resting state. The knockout of CD20 resulted in the translocation of the BCR toward the CD19 coreceptor, transient B cell activation, and internalization of various B cell-specific proteins (10). Additionally, initial research proposed that CD20 may function as a calcium channel (11), however, subsequent findings suggested that calcium flux induction is mediated by the CD20-BCR complex rather than CD20 alone (12). Therefore, the role of CD20 as a regulator of B cell activity seems to be inherently linked to interactions of CD20 with other surface proteins, primarily with the BCR complex.

The regulation of the *MS4A1* gene was attributed to several transcription factors, summarized in (13). The described positive regulators include transcription factors essential for B cell development and maturation, such as PU.1, PiP (IRF4), NFκB (14–16), as well as other factors, such as USF, TFE3.1 (16), OCT1, OCT2 (17), ELK1, ETS1 (18), SP1, CHD4 and MBD2 (19). Recently, another member of interferon regulatory factors (IRF) engaged in B cell development, IRF8, was shown to promote CD20 expression in DLBCL as well as in healthy B cells (20). Negative regulators of CD20 include FOXO1 (21), CREM (19), SMAD2/3 (22), and MYC (23, 24).

Additional regulation of CD20 expression occurs on the epigenetic level. Histone deacetylase (HDAC) family members HDAC1/2, HDAC1/4, and HDAC6 as well as methyltransferase enzyme EZH2 can repress CD20 in healthy and malignant B cells (25–27). Recently, the occurrence of four 5’-UTR variants of *MS4A1* mRNA with differential translation efficacy was described (28).

## Anti-CD20 mAbs and immunoconjugates

The evolution of anti-CD20 mAbs marks a progression toward enhanced compatibility and reduced immunogenicity. The first therapeutic anti-CD20 mAb, rituximab, comprises a chimeric murine-human structure, contributing to the development of immune response and infusion-related reactions due to its limited resemblance to natural human antibodies (29, 30). Enhanced human content in subsequent mAbs correlates with decreased immunogenicity and improved binding affinity to human Fc receptors. The newer generations of anti-CD20 mAbs, exhibiting humanized (obinutuzumab) and fully human (ofatumumab) designs have reduced immunogenicity (31–33). Furthermore, heightened human sequence content enhances interactions with immune effector cells and FcRn receptors on hepatic and epithelial cells, thereby prolonging IgG antibodies’ half-life (34).

CD20-targeting mAbs elicit their cytotoxic function by at least four different mechanisms (35). Upon binding CD20 on target cells, they can activate complement-mediated cytotoxicity (CDC), engage immune effector cells to mediate antibody-dependent cytotoxicity (ADCC) and phagocytosis (ADCP), as well as directly induce cell death. The anti-CD20 mAbs currently employed in cancer treatment vary in their degree of activating specific mechanisms. These differences are the basis for a categorization of anti-CD20 mAbs into two types. The majority of mAbs are characterized as type I, which exhibit the ability to cluster CD20 into membrane lipid rafts, which is associated with potent induction of CDC. On the other hand, type I antibodies display a higher rate of internalization, which can limit their therapeutic efficacy (36). Type II mAbs do not stabilize CD20 in lipid rafts and are weak inducers of CDC, but they potently evoke direct cell death (37). Rituximab, ofatumumab, and ublituximab are classified as the type I anti-CD20 mAbs, whereas obinutuzumab represents a type II anti-CD20 mAb.

CD20-directed mAbs are also used in the form of immunoconjugates – Abs linked with drugs (ADCs), toxins (immunotoxins or engineered toxin bodies – ETBs) or radioactive isotopes (radio-immunoconjugates). Unlike conventional antibodies, which largely depend on the immune effector cells or the complement system for their cytotoxic effects, immunoconjugates can directly induce apoptosis of cancer cells. Due to the relatively poor internalization of CD20, few ADCs and immunotoxins were developed. A single-chain variable fragment-based targeting CD20 and conjugated with Shiga-like toxin A subunit, MT-3724, presented promising preclinical and early clinical results, but its development was ceased by the manufacturer (38). Another strategy that does not require CD20 internalization for direct and targeted cell killing is the use of radio-immunoconjugates. This approach has gained significant attention in targeting lymphoma cells, which are highly radiosensitive (39). Radio-immunoconjugates utilize ionizing radiation to induce cytotoxicity of the target cell. Concurrently, they can trigger classical effector mechanisms such as CDC, ADCC, and ADCP.

In this section, we describe anti-CD20 mAbs and conjugates that were approved for clinical use in lymphoid malignancies. Additionally, other anti-CD20 agents that displayed effectiveness in clinical trials of B-cell neoplasms are listed in **Table 2**.

### Rituximab

Rituximab is the first mAb used for cancer therapy. It is a chimeric mouse/human IgG1 anti-CD20 mAb targeting the epitope on a large extracellular loop of CD20. As a type I mAb, rituximab elicits its function mostly by CDC, ADCC, and ADCP (35). Since gaining its first approval for low grade FL in 1997 (40), rituximab has consistently demonstrated its efficacy, both as part of combination drug regimens and as a standalone agent, across various clinical trials. Rituximab is currently employed in a broad spectrum of conditions including DLBCL, Burkitt lymphoma (BL), MCL, FL, marginal zone lymphoma (MZL), hairy cell leukemia (HCL), and CLL, as comprehensively reviewed in (41). A relatively recent hematologic application of rituximab involves its use in CD20^+^ adult B-ALL, serving as an adjunct to chemotherapy throughout all stages of treatment (7). The popularity and effectiveness of rituximab, as well as the expiration of its patent, has catalyzed an increase in the production of biosimilars. Following prior studies confirming its bioavailability, a new formulation of rituximab with hyaluronidase has been approved for subcutaneous use in FL, CLL, and DLBCL (42). Despite the success of rituximab, some patients experience relapses due to various resistance mechanisms, including trogocytosis, complement exhaustion, internalization of CD20 and others, described in the section *Resistance to CD20-directed immunotherapies* (43, 44). Attempts to increase the efficacy of rituximab prompted the trials combining rituximab with other drugs that could potentiate its cytotoxicity, ideally in chemotherapy-free schemes. A phase 3 study AUGUMENT confirmed the benefit of the addition of the immunomodulatory drug lenalidomide to the rituximab in r/r FL and MZL (45). Strategies involving the addition of mTOR inhibitors to rituximab combined with classic chemotherapeutics are also under investigation for the treatment of patients with r/r DLBCL, with promising results from phase 1 and 2 trials (46, 47).

### Ofatumumab

Ofatumumab (2F2) is a fully human anti-CD20 IgG1κ mAb developed by Genmab and Glaxo PLC. It binds to an epitope distinct from that of rituximab, targeting both small and large extracellular loops of CD20 (48). Preclinical tests have shown that ofatumumab induces CDC more potently than rituximab, while the ADCC efficacy is comparable to that of rituximab (49, 50). The superior CDC efficacy of ofatumumab may be in part associated with the location of its target epitope more proximally to the cell membrane than the epitope recognized by rituximab (51). Recent structural studies also revealed that ofatumumab complexes show optimal geometry for complement recruitment (52). Additionally, ofatumumab demonstrates a slower off-rate than rituximab (49), allowing prolonged binding to the target cells. The first approval of ofatumumab was granted in 2009 for refractory CLL. Despite promising preclinical results, there is limited clinical evidence to confirm its superiority over other anti-CD20 agents (53). Clinical trials comparing ofatumumab to rituximab in FL (54) and DLBCL (55) relapsed after a rituximab-containing therapy showed no superiority of ofatumumab. On the other hand, the comparison of the treatment composed of hyper-fractionated cyclophosphamide, vincristine, doxorubicin, dexamethasone with ofatumumab (HCVAD-O) to the historical cohort of B-ALL CD20^+^ Ph^-^ patients treated with HCVAD with rituximab (HCVAD-R) showed improvement in event-free survival (EFS) and overall survival (OS) (56). Currently, ofatumumab is rarely used in its initial indication, being replaced by newer agents such as obinutuzumab or ibrutinib (57–59).

### Obinutuzumab

Obinutuzumab (GA101) is a humanized, glycoengineered IgG1 type II mAb that targets the epitope on the large extracellular loop of CD20, which partially overlaps with the rituximab epitope. The novelty of obinutuzumab design lies predominantly in the glycoengineering modifications, which were applied to improve affinity to the FcγR receptors on effector cells (60). Specifically, obinutuzumab exhibits reduced fucosylation of oligosaccharides attached to Asp297 in its Fc region, which results in improved binding of FcγRIII (61). In preclinical tests, obinutuzumab presented a slower internalization rate after binding to CD20 and superior efficacy in ADCC than rituximab and ofatumumab (62). The ADCP efficacy was comparable between the three antibodies (63). As a type II mAb, it exhibits reduced levels of CDC (60, 63), but was suggested to have the ability to induce direct cell death (DCD) via a non-apoptotic, lysosome-mediated mechanism, in some, but not all target cell types (64, 65). While obinutuzumab has consistently demonstrated greater effectiveness than equivalent doses of rituximab in the preclinical *in vivo* models (60, 66, 67), the exact reasons behind this advantage remain incompletely understood. The underlying mechanism appears to be multifaceted and potentially attributable to the combination of several factors including greater induction of ADCC and DCD, as well as being less prone to internalization (63). Importantly, obinutuzumab demonstrated superior efficacy as a part of the chemotherapy regimen in comparison with the same chemotherapy but with rituximab in first-line treatment of CLL patients, demonstrating improved progression-free survival (PFS) and OS in a phase 3 trial (68). This resulted in the approval of obinutuzumab in combination with chlorambucil for the treatment of patients with previously untreated CLL in 2013 (69). Recently published results from the phase 3 trial have also demonstrated the benefit of obinutuzumab over rituximab when used as a part of immunochemotherapy in the first-line treatment of FL (70). On the other hand, no advantage over rituximab was observed in advanced DLBCL (9, 71, 72). It is also important to note that the overall doses of obinutuzumab in the clinical trials were higher for most patients (68, 70, 73). An ongoing trial will assess the efficacy of obinutuzumab versus rituximab in B-ALL (NCT04920968). Additionally, promising results of the phase 1 trial of the combination of obinutuzumab with the novel oral cereblon-modulating agent avadomide suggest the potential for new chemotherapy-free regimens for NHL (74). Comprehensive information about obinutuzumab and its efficacy is reviewed in (69, 73, 75).

### Radio-immunoconjugates: 90-Y-Ibritumomab tiuxetan and 131I-Tositumomab

Y-90-Ibritumomab tiuxetan is a murine anti-CD20 IgG1 mAb linked with Y-90 isotope of yttrium, which emits beta radiation and decays to non-radioactive Zirconium-90. A randomized controlled trial of 90-Y ibritumomab tiuxetan in r/r low-grade, follicular, or transformed NHL showed a significant improvement in overall response rate (ORR) and complete response (CR) rates (ORR 80% vs. 56%; CR 30% vs. 16%) in comparison to the rituximab treatment (76). 90-Y ibritumomab tiuxetan was approved in 2002 for r/r NHL patients, and in 2014 the approval was expanded for the first-line consolidation in NHL (77, 78).

131I-Tositumomab is a murine anti-CD20 monoclonal IgG2 antibody linked with Iodium-131, which emits beta and gamma radiation. It was approved for use in r/r NHL in 2003. Despite the documented efficacy in FL and r/r NHL (79, 80) 131I-Tositumomab was replaced by modern agents and its sale was discontinued in 2014.

While the use of radio-immunoconjugates is linked to an increased risk of secondary malignancies and myelotoxicity, their overall toxicity profile was considered acceptable and comparable to other therapies (39, 79, 81–84). Nonetheless, neither of the two radio-immunotherapeutic agents has been widely used in clinical practice, mainly due to economic and logistic problems, such as radiation safety concerns (85). Radio-immunotherapeutics targeting CD20 are extensively reviewed in (86).

### Side effects of anti-CD20 mAbs and their management

The toxicity of anti-CD20 mAbs is relatively low, with hypersensitivity reactions, myelosuppression, and immunosuppression being the most common. Other common side effects include chest pain, arrythmia, paresthesia, nausea, diarrhea, abdominal pain, and muscle pain (87, 88). Rarely, more severe complications may occur, including tumor lysis syndrome or progressive multifocal leukoencephalopathy (PML). Common strategies for reducing hypersensitivity reaction incidence include premedication by steroids or antihistamine drugs and a slow rate of first infusion (89). For CLL patients with high lymphocyte counts (over 25 x 10^9/L), administration of *i.v.* prednisone or prednisolone is recommended before the infusion of rituximab to decrease the risk of acute infusion reactions and/or cytokine release syndrome (CRS) (88). Additionally, a recent study confirmed that obinutuzumab - as a humanized and potentially less immunogenic antibody - can be used as an alternative to rituximab after a hypersensitivity reaction (31). In the case of hypogammaglobulinemia, intravenous immunoglobulin (IVIG) replacement should be considered to reduce the risk of infections (90). Radiolabeled antibodies exhibit additional toxicities related to the emitted radiation, including the risk of myelodysplastic syndrome (MDS) and acute myeloid leukemia (AML) (86). Additionally, the use of 131I-Tositumomab can lead to hypothyroidism. This was addressed by oral administration of potassium iodide to inhibit the thyroid uptake of Iodium-131 (86).

## CD20-directed BsAbs

BsAbs represent one of the most promising classes of off-the-shelf immunotherapies for the treatment of r/r B cell malignancies (91, 92). Notably, several BsAbs targeting the CD20 antigen received clinical approvals in 2023, with numerous others showing promising results in ongoing clinical trials (**Figure 2**, **Table 3**) (93). These engineered proteins, featuring dual binding sites, can simultaneously target two different antigens or two epitopes of the same antigen. This dual specificity allows BsAbs to bridge immune cells, such as T cells, with target tumor cells, promoting their interaction and subsequent cytotoxicity against the tumor cells.

Over the past few years, there has been a rapid development of this technology, resulting in various molecular BsAb formats, including IgG-like and non-IgG-like platforms (91, 92). IgG-like BsAbs mimic the structure of IgG, featuring an Fc region for effector functions, like ADCC and CDC, and provide a larger molecular weight, which increases solubility, stability, serum half-life. This allows for a wider spectrum of dosing frequency from daily to weekly or even less frequent and its administration both intravenously and subcutaneously. In contrast, non-IgG-like BsAbs lack the Fc region, typically have a smaller molecular weight, and primarily exert therapeutic effects through direct antigen binding (92). This applies also to blinatumomab, the first BsAb approved for medical use. Blinatumomab is a CD3xCD19 bispecific T-cell engager that consists only of two scFvs connected by a linker, which contributes to a relatively short half-life and the necessity for frequent dosing in prolonged infusions (94).

Among the various formats, the anti-CD20xCD3 BsAb engaging cytotoxic T cells is the most popular format. Here we focus on CD20-targeting IgG-like BsAbs as a new therapeutic option for patients with B-cell malignancies who have already undergone several lines of mAbs and CD19 CAR-T therapy.

### Epcoritamab (DuoBody-CD3xCD20, GEN3013)

It is a full-length IgG1 BsAb generated by Fab-arm exchange of a humanized CD3 mAb and human CD20 mAb (95). In preclinical studies, epcoritamab has demonstrated its efficacy by eliciting robust T-cell activation and T-cell-mediated cytotoxicity against NHL cell lines *in vitro* (95). It also showed high effectiveness against primary cells derived from lymph node biopsies from newly diagnosed and r/r B-NHL patients (96). Moreover, epcoritamab-mediated cytotoxicity was observed against primary CLL cells *in vitro* and *in vivo* in patient-derived xenografts (PDX), where epcoritamab demonstrated a reduction in both blood and spleen disease burden. This effect was enhanced when used in combination with Bruton’s tyrosine kinase (BTK) and BCL2 inhibitors (97).

These promising results led to the testing of epcoritamab in clinical trials. In the first-in-human trial in patients with r/r B-cell lymphoma, including DLBCL, FL, MCL, high-grade B-cell lymphoma (HGBCL), primary mediastinal large B-cell lymphoma (PMBCL), small lymphocytic lymphoma (SLL) and MZL (EPCORE™ NHL-1, NCT03625037), epcoritamab administered as a single agent subcutaneously in 68 patients exhibited notable efficacy (88% ORR and 38% CR at 48 mg) (98). In an ongoing clinical trial evaluating the safety and efficacy of epcoritamab in patients with r/​r CLL and Richter’s syndrome (EPCORE™ CLL-1, NCT04623541) so far epcoritamab was well tolerated (99). Several other clinical trials using this BsAb are currently underway, including testing a combination with rituximab for the first-line FL (NCT05783609). In 2023 the encouraging outcomes of clinical trials resulted in FDA and EMA approval of epcoritamab for r/r DLBCL after at least two lines of systemic therapy (100, 101).

### Odronextamab (REGN1979)

This hinge-stabilized, fully human IgG4 BsAb targeting CD20 and CD3, has demonstrated both *in vitro* and *in vivo* efficacy (102, 103), leading to further evaluation of its effectiveness in clinical trials. In a phase 1, multicenter trial (ELM-1, NCT02290951) investigating the safety and tolerability of odronextamab in 145 patients with CD20^+^ B-NHL pretreated with CD19 CAR-T therapy or refractory to the last line of therapy, ORR among all patients reached 51% (72 of 142 patients), but among those with FL who received doses of 5 mg or higher 91% ORR (29 of 32 patients) and 72% CRR (23 of 32 patients). DLBCL patients who received doses of 80 mg or higher without previous CAR T-cell therapy reached 53% ORR and all responses were complete, and those pretreated with CAR T-cell 33% ORR and 27% CR (104). Additionally, the efficacy and safety of odronextamab were demonstrated in a case report of two patients with r/r B-NHL refractory to CAR-T therapy, who achieved complete responses that persisted for over 2 years of follow-up (105). In the light of these results, currently the phase 2 clinical trial is conducted. It assesses the anti-tumor activity and safety of odronextamab in pretreated patients with B-NHL (ELM-2, NCT03888105).

Odronextamab is not yet fully approved for marketing, but in September 2023 FDA accepted it for Priority Review for the treatment of adult patients with r/r FL and r/r DLBCL after at least two prior systemic therapies (106). Almost at the same time, EMA has accepted it for review in the same medical indications (107). Previously this drug was designated by EMA as an orphan drug for FL and DLBCL.

### Mosunetuzumab (BTCT4465A)

It is a full-length, humanized IgG1 CD20xCD3 BsAb, generated using “knobs-into-holes” heterodimerization technology, which allows the combination of two heavy chains, one with the ‘knob’ mutation and the other with the ‘hole’ mutation, into one BsAb (108, 109). It was effective *in vitro* against tumor B cells obtained from PBMC of CLL patients, and *in vivo* in mice and cynomolgus monkeys, causing complete B cell depletion in peripheral blood and lymphoid tissues also in the presence of a competitive anti-CD20 mAb (108).

It has been tested in phase 1/2 clinical trial verifying it as a single agent and combined with atezolizumab (anti-PD-L1 mAb) in NHL and CLL (NCT02500407) and demonstrated notable efficacy and a manageable safety profile in patients with r/r FL (ORR 78%, CR 60%) (110). In patients with r/r DLBCL, including those previously treated with CAR-T cells, ORR was 42% and CR 23.9% (111). It is also being investigated in combination with polatuzumab vedotin (CD79b-directed ADC approved for patients with previously untreated DLBCL, NOS and HGBL with International Prognostic Index (IPI) score of at least 2) in B-NHL (NCT03671018) where it shows a favorable safety profile with highly durable responses (112). Currently, many other single-agent and combination studies of mosunetuzumab in r/r and previously untreated B-NHL are ongoing. In June 2022 mosunetuzumab obtained conditional approval from EMA (113) and in January 2023 FDA approved it for adult patients with r/r FL after two or more lines of systemic therapy (114).

### Glofitamab (RO7082859)

It is CD20xCD3 heterodimeric human IgG1 BsAb with two anti-CD20 and one anti-CD3 Fabs (115). In preclinical studies, it showed higher potency than classical 1:1 IgG BsAbs, and its main treatment-related risk of CRS was mitigated by prior treatment with obinutuzumab (115). This combination of anti-CD20 therapies was evaluated in phase 1/2 clinical trial in patients with r/​r B-NHL (NCT03075696), where it demonstrated durable responses, with most patients in CR (116, 117). These clinical findings were also confirmed in a group of 46 heavily pretreated patients with r/r DLBCL, who were given the drug under compassionate use and reached 7 months median OS (118). Glofitamab is also tested in combination with polatuzumab vedotin plus rituximab, cyclophosphamide, doxorubicin and prednisone (R-pola-CHP) and shows promising early results (ORR 100% and CR 76,5% among 17 patients) (119). Therefore in June 2023 FDA granted accelerated approval to glofitamab for r/r DLBCL, not otherwise specified (NOS) or large B-cell lymphoma (LBCL) arising from FL, after two or more lines of systemic therapy (120), and in July EMA approved it for conditional use for adults with r/r DLBCL after at least two previous treatments (121).

### Imvotamab (IGM-2323)

This CD20xCD3 IgM BsAb is generated from 10 high-affinity CD20 binding domains and a single anti-CD3 scFv fused through the recombinant J-chain (122, 123). It exhibits a higher avidity for the CD20 binding and induces CDC against CD20-expressing cells with a greater potency than IgG BsAbs *in vitro* (122). Moreover, it exhibits vastly reduced cytokine release *in vitro* and *in vivo* (122) and seems to maintain higher effectiveness in the presence of rituximab than IgG BsAbs (124). A combination of imvotamab and loncastuximab tesirine (CD19-directed ADC approved in r/r LBCL after two or more lines of systemic therapy) demonstrated enhanced cytotoxic effect in preclinical studies (125) and is currently tested in first-in-human clinical trial in patients with r/r NHL (NCT04082936). So far imvotamab shows notable safety and tolerability profile due to repeatable IFNγ-dominant cytokine profile (123, 126).

### Plamotamab (XmAb13676)

This humanized CD20xCD3 IgG1 BsAb is heterodimer with one IgG Fab arm exchanged for a scFv (127, 128). Preclinical *in vivo* data show its efficiency both in circulation and lymphoid organs (127). A phase 1 clinical trial (NCT02924402) evaluating its safety and tolerability in patients with CD20-expressing hematologic malignancies is ongoing and demonstrated so far evidence of clinical activity in heavily pretreated patients with DLBCL and FL, including earlier treatment with CAR-T therapy (129, 130).

### Other BsAbs

There are several new directions in the further development of BsAbs, involving the use of antigens other than CD3. These include, among others, CD20xNKG2D antibodies, which engage the cytotoxic activity of NK cells against leukemic cells *in vitro* (131, 132). Another novel type of BsAb tested in preclinical studies is the CD95xCD20 antibody, which induces apoptosis in malignant B cells both *in vitro* and *in vivo* (133, 134). Finally, CD20xCD28 antibodies, which were first created over 20 years ago, however, due to the high production costs using conventional methods, were not developed for a long time (135, 136).

### Side effects of CD20-directed BsAbs

Although significant therapeutic successes have been observed in clinical trials, CD20xCD3 BsAbs are associated with certain side effects. The most common is CRS, primarily associated with the initial doses and confined to the first cycle of treatment (110, 137). This is related to the simultaneous binding of BsAb to CD3 of effector cells and FcγR of other immune system cells or complement factor C1q, which results in premature activation and release of cytokines, hampering the effectiveness of therapy and increasing its toxicity. Therefore, currently used BsAbs have silencing mutations in the Fc regions that prevent binding to FcγR and C1q but retain binding to FcRn, which ensures extended plasma half-life (138). Another strategies to overcome CRS are step-up dosing of BsAbs (111, 139) and premedication with anti-CD20 mAb, which depletes B-cells in both peripheral blood and secondary lymphoid organs and decreases T cells activation (115). Other common adverse events include pyrexia, fatigue, injection-site reaction, nausea, diarrhea, hypophosphatemia, hematological toxicities: neutropenia, anemia, lymphopenia, thrombocytopenia, as well as neurological adverse events: headache, insomnia, dizziness (98, 99, 104, 110–112, 117–119, 126, 129, 130, 137, 139–143). Immune effector cell-associated neurotoxicity syndrome (ICANS) is a rare complication that occurs in less than 5% of patients treated with BsAb (119, 137, 139). The frequency and severity of these side effects vary. To mitigate risks, careful patient monitoring, premedication with anti-CD20 mAb, and dose adjustments are implemented to enhance the safety profile of CD20xCD3 BsAb therapies.

## CD20-directed CAR-T cells

CD20 is also under exploration as a target for CAR-T cells in preclinical and clinical trials. CARs are synthetic constructs comprising extracellular antigen recognition domains, hinge and transmembrane regions, and intracellular signaling domains responsible for their activation and proliferation. Approved CAR T-cell therapy involves genetically engineered autologous products, utilizing the patient’s CAR T cells to target tumor cell antigens. Currently, four CD19-targeted CAR T-cell therapies are approved for treating r/r B-ALL and r/r B-NHL. Despite its efficacy, around 60% of patients experience disease relapse post-CD19 CAR-T treatment, often due to mechanisms like CD19 antigen loss. Also, in some patients, life-threatening toxicities occur, including severe CRS and ICANS (144, 145). Ongoing clinical trials suggest that CD20 CAR T-cell therapy could be a promising treatment for r/r NHL, even in cases of CD19-negative disease post-CD19 CAR-T cell relapse (**Table 1**). The structure of the clinically tested CD20 CAR T-cells is presented in **Figure 3**.

Phase 1/2 clinical trials utilizing second- and third-generation CAR constructs have confirmed the feasibility and efficacy of autologous anti-CD20 CAR-T cells in r/r CD20^+^ B-NHL (146). Particularly noteworthy is the efficacy of CD20 CAR T-cell therapy in treating r/r B-NHL patients who had previously failed chemotherapy, including R-CHOP. Studies indicate that CD20-targeted CAR T cells exhibit effectiveness even in cases of low antigen expression, proposing their potential utility for patients with CD20-downregulated B-NHL refractory to CD20 mAb therapy (28, 147). A comprehensive overview of ongoing and completed clinical trials for single CD20 CAR T-cell therapy in hematologic malignancies can be found in Table 2 of a recent review (148).

CD20 is also a pivotal target in CAR-T cell immunotherapies designed to mitigate antigen escape risks. Strategies targeting both CD19 and CD20 include bispecific/tandem CARs, co-administration of CD19 and CD20-directed CAR-T cells as well as sequential treatment with CD19 and CD20-directed CAR-T cells. Tan CAR7 T cells are bispecific CAR T cells composed of tandem extracellular domains targeting CD20 and CD19 tumor antigens linked in frame to the tisa-cel backbone, capable of activation via binding to either CD19 or CD20 tumor antigens, or both (149). Long-term remissions were observed following the use of Tan CAR7 T cells in r/r B-NHL with a safety profile that included CRS but few cases of high-grade CRS (150, 151). In a recent phase 1 dose-escalation trial, autologous CD19/CD20 bispecific CAR-T cells derived from naïve and memory T cells demonstrated safety and strong efficacy (90% ORR, 70% CR rate) in patients with r/r B-NHL (152). Beyond bispecific CD19/CD20 CAR T-cells, ongoing clinical trials explore sequential CD20 CAR-T after CD19 CAR-T infusion and combined infusion of CD19 and CD20-specific CAR-T cells for r/r B-ALL or DLBCL. However, a phase 2 trial combining anti-CD19 and anti-CD20 CAR-T cells in r/r DLBCL showed limited long-term responses (153). Recently, a combinatorial CAR-T cell approach targeting three antigens, CD19, CD20, and CD22, demonstrated efficacy in preclinical models, including leukemic cells that do not express CD19, thereby showcasing the promising potential for treating CD19-negative relapses (154). This approach is now undergoing testing in a clinical trial (NCT05418088).

## Resistance to CD20-directed immunotherapies

Despite substantial progress in CD20-targeting immunotherapies, the issue of resistance and post-treatment relapse remains prominent. Resistance to CD20-targeted therapies encompasses a spectrum of mechanisms, ranging from alterations in CD20 antigen levels to compromised immune system effector functions, and extending to diverse mechanisms of immune evasion (**Figure 4**). One of the main causes of resistance is the loss of the CD20 antigen on the surface of the target cell, which can be caused by changes in the expression of the *MS4A1* gene, including silenced expression and alternative splicing (28, 155–158). A recent study has shown that the gene encoding CD20 in both healthy and malignant B cells is alternatively spliced into four 5’-UTRs variants, of which especially variants V3 and V4 support robust translation. It has also been demonstrated that resistance to the BsAbs therapy targeting CD20 results from the V3-to-V1 shift (28). A potential strategy to combat this resistance through the use of phosphorodiamidate morpholino oligomers or antisense oligonucleotides was presented in preclinical studies (28). Other mechanisms of antigen loss which may also cause resistance include the internalization of the CD20-mAb complex by cancer cells through endocytosis as well as the transfer of membrane fragments containing CD20 from a cancer cell to an effector cell called trogocytosis (159–161).

Resistance to CD20-directed immunotherapies may also be caused by impaired effector functions of the immune system, such as CDC and ADCC. Therapies targeting CD20 may cause complement depletion and overexpression of its inhibitors CD55 and CD59 (44, 162). Additionally, downregulation of the complement component C1qA was associated with the resistance of DLBCL cells to rituximab *in vitro* (163). Potential strategies to overcome these mechanisms may include the use of inhibitors of complement regulatory proteins as well as the use of the new asymmetric CD55-binding bispecific antibodies (164). Moreover, rituximab-coated tumor cells were shown to significantly downregulate CD16 (FcγRIII), leading to impaired ADCC (165). Mutations that modify the binding of the Fc fragment of antibodies to FcγR can be used to increase the effector functions of antibodies (166, 167). However, as previously mentioned, this approach may not always be optimal when utilizing BsAbs, as it carries an increased risk of premature activation of T cells, cytokine release, and tissue damage.

Additionally, genetic alterations within signaling pathways can also contribute to resistance to CD20-directed therapies, especially in the context of T cell activation, which is crucial for the activity of BsAbs and CAR-T cells (168, 169). Moreover, the tumor microenvironment can play a role in resistance by creating an immunosuppressive milieu. Tumor cells and immunosuppressive cells in the tumor microenvironment, e.g. myeloid-derived suppressive cells (MDSCs), tumor-associated macrophages (TAMs), and regulatory T cells (Tregs), can secrete suppressive cytokines that inhibit the activity of effector cells (T cells, NK cells, phagocytes), thereby reducing the effectiveness of immunotherapy (170, 171). It has also been shown that some proteins secreted by the tumor may have a suppressive effect, including galectin-1, which inhibited CD20 mAb-induced phagocytosis in the lymphoma microenvironment (172). Moreover, overexpression of PD-L1 by tumor cells can contribute to resistance to CD20-targeting therapies by dampening the activity of effector T cells induced by these therapies. Tumor cells may increase PD-L1 expression in response to treatment, leading to T cell exhaustion and reduced efficacy of CD20-targeting therapies (173). Combining CD20-targeting therapies with immune checkpoint inhibitors is a potential strategy to overcome resistance and is currently tested in clinical trials, as discussed in more detail in section *Combination therapies with CD20 immunotherapeutics.*

Due to the multitude of resistance mechanisms, it is crucial to actively search for new methods that can increase the effectiveness of immunotherapy. Research efforts encompass the identification of novel therapeutic targets beyond CD20, the refinement of patient stratification, and the incorporation of combination therapeutic strategies. A recent review summarizes potential solutions to overcome resistance to CAR-T therapy (174).

## Combination therapies with CD20 immunotherapeutics

To enhance their efficacy, anti-CD20 mAbs are commonly administered in combination with other drugs. One notable combination is the R-CHOP regimen, which integrates rituximab with cyclophosphamide, doxorubicin, vincristine, and prednisone, and has been extensively employed in treating patients with DLBCL and MCL. Similarly, R-pola-CHP (rituximab, polatuzumab, cyclophosphamide, doxorubicin) regimen is an approved treatment for advanced-stage DLBCL. Other regimens include a combination of rituximab, dexamethasone, high-dose cytarabine and a platinum-based agent (R-DHAP) used in the treatment of MCL, and the addition of lenalidomide to rituximab (R-lenalidomide) which has shown promising results, particularly in patients with r/r FL. Combinations such as bendamustine and rituximab (BR) and fludarabine, cyclophosphamide, and rituximab (FCR) have demonstrated efficacy in the treatment of CLL. Furthermore, novel combinations of rituximab with targeted agents have shown significant potential. Rituximab in combination with venetoclax, a BCL-2 inhibitor, as well as with idelalisib, a PI3K inhibitor, has been approved for the treatment of CLL. Furthermore, the R-GemOx regimen, which combines rituximab with gemcitabine and oxaliplatin, has exhibited notable efficacy in r/r B-NHL in phase 2 clinical trial (175), and is currently being compared to a similar regimen using glofitamab instead of rituximab (glofit-GemOx) in a phase 3 clinical trial (176). In the treatment of CD20^+^ B-ALL, rituximab is added to standard chemotherapy regimens in patients with a Philadelphia chromosome-negative (Ph-) B-ALL. In patients with Philadelphia chromosome-positive (Ph+) CD20^+^ B-ALL, rituximab is combined with chemotherapy and BCR-ABL1 tyrosine kinase inhibitors, such as imatinib and dasatinib.

Several clinical trials have investigated the efficacy of other combination therapies involving CD20-targeting in patients with B-NHLs. Among these trials, the combination of R-DHAP regimen with temsirolimus, an mTOR inhibitor, has shown encouraging results, demonstrating improved outcomes in patients with r/r DLBCL (47). Temsirolimus has also demonstrated effectiveness in combination with rituximab alone in patients with r/r MCL in phase 2 clinical trial (177). Moreover, checkpoint inhibitors are also being tested in phase 1 and 2 clinical trials in combination with anti-CD20 therapies. The addition of atezolizumab to an R-CHOP regimen in previously untreated DLBCL patients resulted in 77,5% CR (178). It is also tested in combination with BsAbs glofitamab and mosunetuzumab in phase 1/2 clinical trials in patients with NHL (NCT02500407, NCT03533283) (179). Additionally, pembrolizumab (anti-PD1 mAb) was evaluated in combination with rituximab in a single-arm phase 2 clinical trial and resulted in 67% ORR with 50% CR among patients with r/r FL (180).

Notably, drugs that are used in combinations with CD20-targeting immunotherapies may have a bidirectional impact on CD20 antigen expression and thus the effectiveness of these therapies. Prednisolone, a glucocorticosteroid present in many chemotherapeutic regimens, was shown to upregulate CD20 on some primary B-ALL samples *in vitro* (4). On the other hand, some drugs that are used together with anti-CD20 mAbs, such as BTK inhibitor ibrutinib, PI3Kδ inhibitor idelalisib, or SYK inhibitor dasatinib, were shown to downregulate CD20 and demonstrated inhibitory effects on cytotoxic effector cells (181–184). These drugs decreased the efficacy of anti-CD20 mAbs *in vitro* (183, 184). It may also be one of the reasons for the failure of an attempt to improve ibrutinib efficacy in CLL by the addition of rituximab, as demonstrated in a randomized clinical trial showing no improvement in PFS in the rituximab+ibrutinib group versus ibrutinib alone (185). This highlights the need for further research on the drug-induced changes in cellular signaling and related CD20 regulation. Understanding these relationships may be important for selecting the most effective therapies and improving therapeutic results. Interestingly, several classes of CD20-upregulating drugs were described, including aurora kinase inhibitors, FOXO1 inhibitors, and chromatin modulators, enabling the increase in anti-CD20-mAbs efficacy in preclinical settings (13). Combining these drugs with CD20-targeting therapies could be a potentially valuable strategy to overcome resistance, however, it requires further evaluation in a clinical setting.

## Concluding remarks

A breakthrough in the treatment of B cell malignancies is evident with recent approvals of CD19 CAR-T cells and BsAbs, particularly those targeting CD20xCD3, offering effective treatment and potential cure for r/r patients. Over the past 25 years, CD20, an early target in immunotherapy, has demonstrated remarkable effectiveness. However, the widespread use of cytotoxic T cell-based therapies appeared with new challenges such as treatment-related complications and side effects. Effective management requires the accumulation of comprehensive knowledge and experience, including identifying risk factors for CRS, ICAN, and refining treatment guidelines. These improvements are crucial for the widespread use of these innovative drugs.

With diverse treatment modalities emerging, from naked mAbs to BsAbs and CAR-T cells, understanding determinants of activity and resistance mechanisms for the specific types of treatment are crucial for their optimal selection and clinical efficacy. Decent levels of CD20 are essential for the efficacy of all types of CD20-directed immunotherapies, however, recent preclinical reports emphasize that different types of anti-CD20 therapies require different amounts of CD20 protein on the cell surface to be effective. While a certain level of reduction in CD20 compromises the activity of anti-CD20 mAbs and BsAbs, it may still be adequate for the effectiveness of CD20 CAR-T cells (28). Although the CD20 CAR-T constructs currently being tested in the clinic show great efficacy, further refinements to the CD20 CAR constructs, including changes around the scFv sequence, have shown significant superiority in preclinical models and offer the prospect of even better outcomes for patients (186).

Key directions for CD20 immunotherapy improvement also include combination strategies with small molecule drugs and simultaneous targeting of multiple immunotherapy targets to enhance precision and minimize relapse risks. Simultaneous targeting of CD20 with other antigens like CD19 and CD22 demonstrates efficacy in preclinical models (154) and ongoing clinical trials. Noteworthy, a better understanding of the determinants of response and resistance will be critical for patient selection and future rational combinations.

## Author contributions

AD: Conceptualization, Writing – original draft, Writing – review & editing, Visualization, Investigation. KD: Conceptualization, Writing – original draft, Writing – review & editing, Investigation. MF: Conceptualization, Investigation, Writing – original draft, Writing – review & editing, Funding acquisition, Supervision, Visualization.
